# Methoxsalen and Bergapten Prevent Diabetes-Induced Osteoporosis by the Suppression of Osteoclastogenic Gene Expression in Mice

**DOI:** 10.3390/ijms20061298

**Published:** 2019-03-14

**Authors:** Ju Ri Ham, Ra-Yeong Choi, Hae-In Lee, Mi-Kyung Lee

**Affiliations:** 1 Department of Food and Nutrition, Sunchon National University, Suncheon 57922, Korea; punsu05@nate.com (J.R.H.); fkdud1304@naver.com (R.-Y.C.); 2 Mokpo Marin Food-Industry Research Center, Mokpo 58621, Korea; hich2731@nate.com

**Keywords:** diabetes, bone microarchitecture, bergapten, methoxsalen, osteoporosis

## Abstract

This study evaluated whether bergapten and methoxsalen could prevent diabetes-induced osteoporosis and its underlying mechanism. For 10 weeks, bergapten or methoxsalen (0.02%, *w*/*w*) was applied to diabetic mice that were provided with a high-fat diet and streptozotocin. Bone mineral density (BMD) and microarchitecture quality were significantly reduced in the diabetic control group; however, both bergapten and methoxsalen reversed serum osteocalcin, bone-alkaline phosphatase and femur BMD. These coumarin derivatives significantly increased bone volume density and trabecular number, whereas they decreased the structure model index of femur tissue in diabetic mice. Conversely, tartrate-resistant acid phosphatase 5 (TRAP) staining revealed that these derivatives reduced osteoclast numbers and formation in diabetic bone tissue. Additionally, both bergapten and methoxsalen tended to downregulate the expression of osteoclast-related genes such as receptor activator of nuclear factor kappa-B ligand (*RANKL*), nuclear of activated T-cells, cytoplasmic 1 (*NFATc1*) and *TRAP* in diabetic femurs, with *NFATc1* and *TRAP* expression showing significant reductions. Our data suggest that both bergapten and methoxsalen prevent diabetic osteoporosis by suppressing bone resorption.

## 1. Introduction

The prevalence of diabetes and osteoporosis are increasing worldwide, and these conditions have led to high morbidity and mortality among the elderly [1]. Both type 1 (T1DM) and type 2 diabetes mellitus (T2DM) have been associated with decreased bone strength and increased risk of bone fractures [2]. Diabetes-related osteoporosis is a general metabolic bone disorder that increases the tendency for fractures due to osteopenia, microstructural changes in bone tissue, decreased bone strength and increased friability, which is one of the main complications of diabetes affecting the skeletal system [3]. The mechanisms linking DM to osteoporosis have not been fully explained, but insulin deficiency and dysfunction, obesity and hyperinsulinemia, altered levels of estrogen, leptin and adiponectin and DM-related complications may be associated with impaired bone metabolism and increased risk of fractures [1]. 

**Figure 1:**
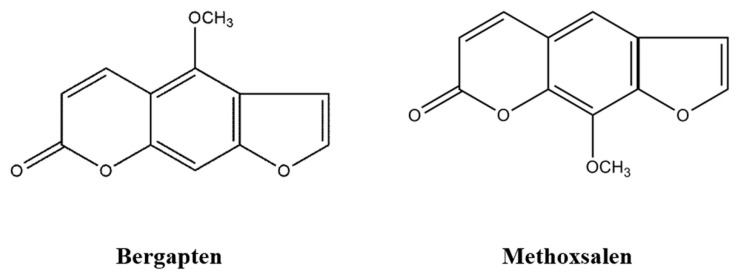
Structures of bergapten and methoxsalen.

Furanocoumarins, which occur in various herbal and citrus extracts, possess antibacterial, antioxidant, immunomodulator, apoptotic and anticancer activities [4]. Bergapten (BP, 5-methoxypsoralen) and methoxsalen (MTS, 8-methoxypsoralen) (Figure 1), two important derivatives of furanocoumarin, are linear furanocoumarins found in natural foods such as celeriac, celery (fresh), lemon, parsley and parsnip [5,6,7,8,9,10]. After oral intake in both humans and animals, furanocoumarins can be absorbed rapidly in the gastrointestinal tract [11,12]. They are metabolized mainly via cytochrome P450-dependent monooxygenase in the liver and transformed by epoxidation, hydroxylation, glucuronide conjugation and the hydrolytic opening of the lactone ring [13,14]. BP and MTS bind to serum albumin [13,15]. BP binds to low-density lipoprotein in serum, while MTS has metabolic activation with subsequent covalent binding of metabolites to the microsomal protein [13]. The main excretion route is the kidney and 5–10% is excreted via feces [13].

BP is known as a natural anti-inflammatory and anti-tumor agent and it has been used to prevent lipopolysaccharide-mediated osteoclast formation, bone resorption and osteoclast survival in vitro [16]. Fang et al. [17] suggested BP as a new strategy for T2DM because it ameliorates insulin resistance by the ER (endoplasmic reticulum)-mediated PI3K/AKT activation pathway in HepG2 cells. BP prevented osteoporosis in high-fat diet-induced insulin resistance with osteoprotegerin (OPG) knockout mice by inhibiting the PI3K/AKT, JNK/MAPK and NF-κB signaling pathways [3]. However, the effects of BP on hyperglycemia-induced osteoporosis are not yet understood. MTS is also a natural photoactive compound found in many plant seeds [18] and a structural isomer of BP, but its anti-osteoporotic function has been relatively less studied compared with BP. We would like to develop BP or MTS as a food ingredient or a food supplement for preventing DM-related osteoporosis. We recently showed the anti-osteoporotic activity of MTS in ovariectomized mice [19]. Therefore, this study was conducted to elucidate the anti-osteoporotic ability of BP and MTS in high-fat diet (HFD) and streptozotocin (STZ)-induced diabetic mice. 

## 2. Results

### 2.1. The Effects of BP and MTS on General Characteristics in Diabetic Mice

Body weight, serum insulin and adiponectin levels in DM decreased compared with the non-diabetic (NC) group, whereas blood glucose and glycosylated hemoglobin (HbA_1c_) levels increased (Table 1). Both BP and MTS did not affect these general characteristics of diabetes (Table 1). Serum calcium (Ca) and inorganic phosphorus (IP) concentrations were significantly lower in DM than in the NC. However, BP significantly increased the IP content and MTS slightly recovered it compared with the DM group (Table 1).

### 2.2. The Effects of BP and MTS on Serum Osteoblast and Osteoclast Markers in Diabetic Mice

**Figure 2:**
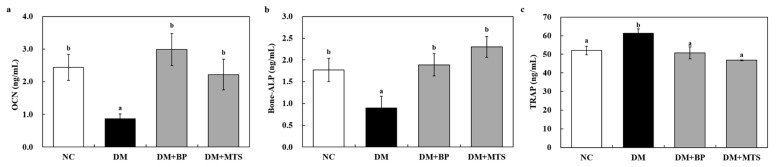
The effects of bergapten or methoxsalen supplementation on serum osteocalcin (OCN) (**a**); bone- alkaline phosphatase (ALP) (**b**) and tartrate-resistant acid phosphatase 5 (TRAP) (**c**) in diabetic mice. Values are expressed as the means ± S.E. (n = 8). ^ab^ Different letters indicate significant differences among experimental groups (*p* < 0.05). NC, normal control group; DM, diabetic control group; DM+BP, diabetic-bergapten group; DM+MTS, diabetic-methoxsalen group.

Serum bone-alkaline phosphatase (ALP) and osteocalcin (OCN) levels were significantly lower in the DM group than in the NC group. However, both BP and MTS significantly increased OCN levels by 3.4- and 2.5-fold, respectively, when compared with the DM group (Figure 2a). Bone-ALP levels were also significantly higher in the BP (2.1-fold) and MTS (2.6-fold) supplementation groups than those of the DM group (Figure 2b). Thus, BP is more efficient in increasing the OCN, whereas MTS is more effective on the bone-ALP level in DM-induced osteoporotic mice. The serum tartrate-resistant acid phosphatase 5 (TRAP) concentration was significantly reduced in both the BP and MTS groups compared to the DM group (Figure 2c).

### 2.3. The Effects of BP and MTS on Bone Microarchitecture and Histology in Diabetic Mice

Femur length did not differ between experimental groups (Table 2). Femur weights were significantly lower in the DM group than the NC group; however, BP significantly increased femur weight whereas MTS supplementation increased femur weight slightly when compared to the DM group (Table 2). Neither the length nor weight of tibia differed among groups (Table 2). 

**Figure 3:**
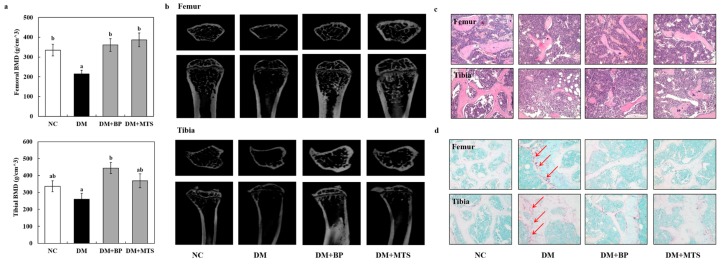
The effects of bergapten or methoxsalen supplementation on bone mineral density (BMD) (**a**); bone micro-computed tomography (μCT) image (**b**); bone hematoxylin & eosin (H&E) staining (**c**) and bone tartrate-resistant acid phosphatase 5 (TRAP)staining (**d**) in diabetic mice. Values are expressed as the means ± S.E. (n = 8). ^ab^ Different letters indicate significant differences among experimental groups (*p* < 0.05). Magnification 200×; red arrows indicate osteoclasts. NC, normal control group; DM, diabetic control group; DM+BP, diabetic-bergapten group; DM+MTS, diabetic-methoxsalen group.

Hyperglycemia significantly decreased femoral bone mineral density (BMD), but both BP and MTS similarly reversed the bone loss. BP also significantly increased the tibial BMD compared to the DM group and showed a slight increase compared with NC and MTS groups (Figure 3a,b). Moreover, DM altered both femoral and tibial trabecular architecture, demonstrating that bone volume density (BV/TV) and trabecular thickness (Tb.Th) were significantly decreased and that structure model index (SMI) was increased in the DM group as compared with the NC group (Table 2). Both BP and MTS similarly increased femoral BV/TV, trabecular number (Tb.N) and Tb.Th, whereas they decreased SMI in diabetic mice. Tibial BV/TV, Tb.Th and SMI also recovered in response to BP supplementation, while MTS only increased Tb.Th compared to the DM group. Trabecular separation (Tb.Sp) in both femur and tibia did not differ among groups (Table 2). 

Upon hematoxylin and eosin (H&E) staining, both BP and MTS were determined analogously to augment the thickness and volume relative to the DM group (Figure 3c). On the other hand, TRAP staining, one of the bone resorption markers, revealed that BP and MTS reduced osteoclast numbers and formation in diabetic bone tissue (Figure 3d).

### 2.4. The Effects of BP and MTS on Bone Metabolism-Related Gene Expression in Diabetic Mice

**Figure 4:**
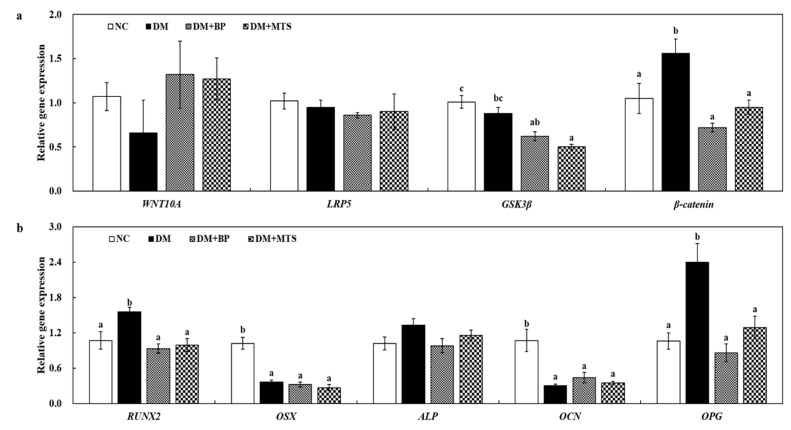
The effects of bergapten or methoxsalen supplementation on the femur Wnt pathway (**a**) and femur osteoblast (**b**)-related gene expression in diabetic mice. Values are expressed as the means ± S.E. (n = 8). ^abc^ Different letters indicate significant differences among experimental groups (*p* < 0.05). NC, normal control group; DM, diabetic control group; DM+BP, diabetic-bergapten group; DM+MTS, diabetic-methoxsalen group.

**Figure 5:**
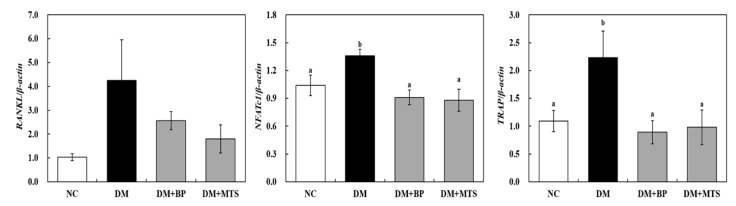
The effects of bergapten or methoxsalen supplementation on the femur osteoclast-related gene expression in diabetic mice. Values are expressed as the means ± S.E. (n = 8). ^abc^ Different letters indicate significant differences among experimental groups (*p* < 0.05). NC, normal control group; DM, diabetic control group; DM+BP, diabetic-bergapten group; DM+MTS, diabetic-methoxsalen group.

To identify the effects of BP and MTS supplementation on bone remodeling, we examined the Wnt pathway, osteoblast and osteoclast-related femoral mRNA expression. DM increased the gene expression of beta-catenin (*β-catenin*), runt-related transcription factor 2 (*RUNX2*), *OPG*, nuclear factor of activated T-cells, cytoplasmic 1 (*NFATc1*) and *TRAP* as well as decreased that of osterix (*OSX*) and *OCN* compared with the NC group (Figure 4 and Figure 5). Both BP and MTS significantly downregulated glycogen synthase kinase 3 beta (*GSK3β*), *β-catenin*, *RUNX2*, *OPG*, *NFATc1* and *TRAP* gene expression compared to DM (Figure 4 and Figure 5). Receptor activator of nuclear factor kappa-B ligand (*RANKL*) was also greatly down-regulated by both BP and MTS, but these differences were not significant (Figure 5). BP and MTS showed similar effects on the changes in gene expression. 

## 3. Discussion

The present study was the first to demonstrate that both MTS and BP supplementation at 0.02% (*w*/*w*) similarly protected against diabetes-induced osteoporosis in mice, which was demonstrated by increased BMD and bone quality. Osteoporosis has a multifactorial etiology and can be postmenopausal, senile or diabetic, and it is characterized by reduced bone mass and poor bone quality, resulting in decreased bone strength with an increased risk of fractures [20,21]. The osteoporotic mechanism in all cases is an imbalance between bone resorption by osteoclasts and bone formation by osteoblasts, which leads to a decreased BMD [20,22]. Diabetic osteopathy is characterized by microarchitectural changes that decrease the bone quality, leading to increased bone fractures in both types of DM [20,23,24]. Previous studies reported that BMD is lower in T1DM, whereas it was normal, low or even high in T2DM relative to that of healthy people [25,26]. In this study, T2DM was induced by combining an HFD (40% kcal from fat) for 4 weeks, which produced insulin resistance, with STZ injection, which caused initial β-cell dysfunction [27]. This model is known to closely mimic not only the phenotype, but also the pathogenesis of human T2DM [28,29]. Since microarchitecture is essential to assessment of bone mechanical properties [30], we conducted quantitative micro-computed tomography (µCT) analysis of the trabecular bone microarchitecture. The results showed that diabetes caused a marked decrease in trabecular BMD, BV/TV, Tb.Th and Tb.N, as well as an increase in SMI that is similar to the changes in estrogen-deficient mice [19]. However, both BP and MTS reversed these changes effectively. Thus, BP and MTS significantly improved the BMD and microarchitecture of trabecular bone in diabetic mice.

OCN, one of the bone formation markers, decreased in DM patients and was inversely correlated with glucose levels [31,32]. Preclinical studies have suggested that OCN stimulates insulin secretion, enhancing energy expenditure and increasing the expression of adiponectin and thus tissue insulin sensitivity [33,34]. In the current study, both BP and MTS significantly elevated the serum OCN and bone-ALP levels that were lowered by diabetes; however, they did not affect blood glucose, HbA_1c_ or serum insulin level when compared to the DM group. These results indicate that the action of BP and MTS on bone loss in diabetic mice may be independent of the regulation of blood glucose level.

To identify how BP and MTS can alleviate diabetes-induced bone loss, we determined their effects on bone metabolism using femur tissue. To maintain bone volume and quality, the differentiation of osteoclasts and osteoblasts is tightly regulated through communication between and within these two cell lineages [35]. The Wnt pathway inhibits osteoclastogenesis by inducing OPG and suppresses bone resorption by an OPG-independent mechanism acting directly on osteoclast precursors [36]. Interestingly, the present study showed that the expression of some critical osteogenesis-related genes in the BP and MTS groups, such as *β-catenin*, *RUNX2* and *OPG*, were close to the values of the NC group, whereas the expression of these genes in the DM group was significantly increased relative to the NC group, indicating that their expression could, in part, be a compensatory response to hyperglycemia-related bone loss under DM. Kiechl et al. [37] reported that OPG concentration was not elevated prior to T2DM onset, but that it increased after disease occurrence in subjects with diabetes, which was consistent with our findings. In addition, we found that *ALP* gene expression in femur tissues was similar to changes in the *RUNX2* gene. A previous study reported that since RUNX2 directly binds to the ALP intron 1, ALP is tightly regulated by RUNX2 [38]. However, we found no obvious alterations in *OSX* and *OCN* expression in either the BP or MTS group. Thus, the effects of BP and MTS on gene expressions of osteoblast activity under hyperglycemia was not fully elucidated in this study.

A previous study reported that hyperglycemia elevated *RANKL* expression, which aggregated osteoclast absorption and osteoporosis [39]. Herein, we found that, although there was no statistical significance, *RANKL* gene expression in DM was 4.2-fold higher than that in the NC group, which was suppressed by BP or MTS supplementation. *RANKL* selectively induces *NFATc1* expression, which is a master switch for regulating the terminal differentiation of osteoclasts [40]. Therefore, the RANKL pathway has been recognized as an effective therapeutic strategy against diabetic osteoporosis [41]. NFATc1 plays a pivotal role in osteoclast fusion and osteoclast activation via the upregulation of various genes responsible for osteoclast adhesion, migration and acidification as well as the degradation of inorganic and organic bone matrix [42]. Our results showed that both BP and MTS significantly downregulated *NFATc1* and *TRAP* gene expressions in diabetic mice. The activation of *NFATc1* induces the transcription of osteoclast-specific genes such as *TRAP*, matrix metallopepetidase-9 (*MMP-9*), osteoclast-associated receptor (*OSCAR*) and cathepsin-K [43,44]. TRAP activity is a marker often used for identifying osteoclasts [45]; therefore, we carried out femoral TRAP staining. The current study confirmed that femoral TRAP staining in the DM group was increased relative to the NC group; however, both BP and MTS effectively recovered TRAP staining, which resulted in decreased serum TRAP levels in these groups compared with the DM group. Taken together, our data suggest that both BP and MTS inhibit osteoclastogenesis via the suppression of *NFATc1* and *TRAP* activation rather than the regulation of osteoblastogenesis in diabetic mice. Thus, BP and MTS could be applicable as a new food supplement or pharmacological agent for the prevention of osteoporosis in diabetics; however, their efficacy and safety need to be supported by clinical trials. 

## 4. Materials and Methods

### 4.1. Materials

Bergapten and methoxsalen (purity > 98%) were purchased from TCI (Tokyo, Japan). STZ was acquired from Sigma-Aldrich (St. Louis, MO, USA). Many kits were purchased; serum bone-ALP and OCN enzyme-linked immunosorbent assay (ELISA) kits from Elabscience (Wuhan, China), a TRAP ELISA kit from Cusabio Biotech (Wuhan, China), an insulin kit from Morinaga Institute of Biological Science, Inc. (Yokohama, Japan) and an adiponectin kit from R&D Systems, Inc. (Minneapolis, MN, USA). TRIzol reagent, ReverTra Ace qPCR RT master mix and SYBR green PCR kit were acquired from Invitrogen (Carlsbad, CA, USA), Toyobo (Osaka, Japan) and Qiagen (Hilden, Germany), respectively. 

### 4.2. Animals

Four-week-old C57BL/6N mice were purchased from Orient Bio Inc. (Seongnam, Korea). The mice were individually housed in polycarbonate cages and kept in a temperature and humidity-controlled environment (22 ± 2 °C, 50 ± 5% humidity) under a 12/12-h light/dark cycle. All experimental protocols involving the use of animals were conducted in accordance with the Institutional Animal Care and Use Committee of Sunchon National University’s guidelines (approval number, SCNU IACUC-2016-11, approval date: 17 November 2016). Mice were fed a pelletized commercial chow diet for 1 week after arrival, then randomly divided into non-diabetic (n = 8) and diabetic (n = 30) groups. Non-diabetic (NC) mice were fed a normal diet (11% calories from fat) and diabetic mice were fed an HFD (40% calories from fat) for 4 weeks, and then injected with STZ (100 mg/kg body weight in 0.1 M citrate buffer, pH 4.2) into the peritoneum on two consecutive days. After 7 days, only STZ-treated mice that exhibited a fasting blood glucose level ≥13.9 mmol/L were used in the study. Diabetic mice were randomly divided into three groups of eight mice each—an untreated diabetic (DM control) group, a diabetic-bergapten (DM + BP) group and a diabetic-methoxsalen (DM + MTS) group. The non-diabetic mice were fed a normal diet, and diabetic mice were fed an HFD for 10 weeks. In the present study, both BP and MTS were added to the HFD diet at 0.02%, which was based on our previous study [19]. The results demonstrated that 0.02% MTS supplementation effectively increased BMD and decreased osteoclast numbers and formation in femur tissues of ovariectomized mice. Body weight was measured weekly and food consumption was measured daily. After 10 weeks, all the experimental mice were fasted overnight and then anesthetized with ether. Whole blood samples were gained from the inferior vena cava under anesthesia conditions and centrifuged at 3000 rpm for 15 min at 4 °C to obtain serum for biomarker analysis. 

### 4.3. Bone Microarchitecture Analysis Using Micro-Computed Tomography (µCT)

The femur and tibia of all mice were obtained, removed and cleaned of adherent soft tissues. They were soaked and stored in 70% ethyl alcohol solution until the scanning. Femoral and tibial lengths were determined using a Vernier caliper (Mitutoyo, Kawasaki, Japan).

The femur and tibia of each mouse were wrapped in plastic wrap to avoid drying and placed in a sample holder. We scanned each femur and tibia using a Skyscan 1272 µCT system (Bruker, Kontich, Belgium) under the following scanning conditions and parameters: 70 kV, 142 µA, 0.5 mm aluminum filter, 26.5 µm resolution, rotation step of 0.4° and triple frame averaging. The reconstruction of the projection images was performed in NRecon software (Bruker, Belgium). The CTAn program (version 1.16.4.1, Bruker, Belgium) was used to quantitatively analyze the data of BMD, BV/TV, Tb.N, Tb.Th, Tb.Sp and SMI. The BMD is a representative bone density parameter. BV/TV, a bone volume fraction, independently suggests how much mineral fill exists in the bone tissue. Tb.N means the average number of trabeculae per unit length. Tb.Th and Tb.Sp define the mean thickness of the trabeculae and mean distance between the trabeculae, respectively. SMI is an indicator of the structure of the trabecular, indicating where it is plate or rod-like [46].

### 4.4. Histological Analysis of Bone Tissue

The distal metaphysis of each femur and tibia was fixed in 10% formaldehyde buffer at 4 °C for histological measurement. The fixed samples were decalcified with 10% ethylenediaminetetraacetic acid (EDTA) buffer. The decalcified samples were embedded in paraffin and sectioned at 3–5 μm, then stained with H&E and TRAP. The stained areas were viewed using an optical microscope at 200× magnification.

### 4.5. Biochemical Analysis

The fasting blood glucose concentration was monitored using a glucometer (G-doctor, AllMedicus, Co., Ltd., Anyang, Korea) to test venous blood drawn from the tail vein every week after a 6-h fast. The HbA_1c_ concentration in whole blood was measured using a NycoCard Reader II (Alere/Axis-Shield, Oslo, Norway). The insulin and adiponectin levels were determined using a quantitative sandwich enzyme immunoassay kit. Serum bone-ALP, OCN and TRAP levels were measured using mouse ELISA kits. Serum Ca and IP levels were determined using an automated blood analyzer (Dri-Chem 3500i: Fujifilm Medical System Co., Ltd., Tokyo, Japan). 

### 4.6. Real-Time PCR (Polymerase Chain Reaction) Analysis

Total RNA was extracted from the femur tissues with TriZol reagent following the manufacturer’s protocols. One microgram of the total RNA was then converted to cDNA using a ReverTra Ace qPCR RT master mix. Next, mRNA expression was measured by RT-qPCR (real-time quantitative polymerase chain reaction) using a SYBR green PCR kit and the CFX96TM real-time system (Bio-Rad, Hercules, CA, USA). The primers used were as follows: *ALP*: forward GTTGCCAAGCTGGGAAGAACAC and reverse CCCACCCCGCTATTCCAAAC; *β-catenin*: forward CAGTGCAGGAGGCCGAG and reverse TCAGGTCAGCTTGAGTAGCC; *GSK3β*: forward TTGGACAAAGGTCTTCCGGC and reverse GGTCCCGCAATTCATCGAAA; *LRP5*: forward GGAGTTCTCAGCCCATCCTT and reverse GTAGGAGGCTCACCACAAGT; *NFATc1*: forward AGGACCCGGAGTTCGACTT and reverse GTCGAGGTGACACTAGGGGA; *OCN*: forward TTTCTGCTCACTCTGCTGACC and reverse CGCCGGAGTCTGTTCACTAC; *OPG*: forward GCCACGCAAAAGTGTGGAAT and reverse TTTGGTCCCAGGCAAACTGT; *OSX*: forward CACCCATTGCCAGTAATCTTCAA and reverse ATAGTGAGCTTCTTCCTGGGTA; *RANKL*: forward CGAGGAAGGGAGAGAACGAT and reverse AGGTACTTGCCGTAGTCTCG; *RUNX2*: forward TACAACTAAAACAGGGACTGGGT and reverse AGGCTGTTTGACGCCATAGT; *TRAP*: forward AGGAAGAGCCTTCAAGTAAGTG and reverse CCACCCATGAATCCATCTTCT; *WNT10A*: forward CGAGGTTTTCGAGAGAGTGC and reverse TTCAGTTTACCCAGAGCGCA; beta-actin (*β-actin*): forward GATCAGCAAGCAGGAGTACGA and reverse GGTGTAAAACGCAGCTCAGTAAC. All of the gene expressions were relatively normalized against the *β-actin* gene.

### 4.7. Statistical Analysis

All data are expressed as the means ± standard error (S.E.). SPSS software was used to analyze all statistical tests (Chicago, IL, USA). Statistically significant differences among the groups were determined by one-way ANOVA with Duncan’s multiple-range test. Values of *p* < 0.05 were considered statistically significant. 

## 5. Conclusions

Both BP and MTS attenuated diabetes-induced osteoporosis via the downregulation of osteoclastic metabolism in mice, but they did not improve hyperglycemia when applied at 0.02% in the diet. Based on these results, BP and MTS can be used in combination with anti-hyperglycemic agents to improve osteoporosis in individuals with diabetes. 

## Figures and Tables

**Table 1:** The effects of bergapten or methoxsalen supplementation on general characteristics in diabetic mice.

	NC	DM	DM+BP	DM+MTS
Initial body weight (g)	26.31 ± 0.41	26.33 ± 0.48	26.22 ± 0.50	26.68 ± 0.36
Final body weight (g)	33.10 ± 0.97 ^b^	24.76 ± 0.48 ^a^	25.79 ± 0.81 ^a^	25.74 ± 1.31 ^a^
Blood glucose (mmol/L)	9.96 ± 0.29 ^a^	27.07 ± 0.98 ^b^	27.22 ± 1.37 ^b^	28.05 ± 1.11 ^b^
HbA_1C_ (%)	4.31 ± 0.08 ^a^	9.64 ± 0.58 ^b^	9.22 ± 0.60 ^b^	9.43 ± 0.29 ^b^
Serum				
Insulin (pg/mL)	742.72 ± 87.26 ^b^	322.78 ± 22.80 ^a^	536.85 ± 92.75 ^ab^	384.31 ± 49.03 ^a^
Adiponectin (μg/mL)	0.93 ± 0.02 ^b^	0.69 ± 0.02 ^a^	0.66 ± 0.03 ^a^	0.67 ± 0.06 ^a^
Ca (U/L)	8.55 ± 0.21 ^b^	7.57 ± 0.30 ^a^	7.72 ± 0.25 ^a^	7.75 ± 0.11 ^a^
IP (U/L)	9.01 ± 0.52 ^c^	5.32 ± 0.45 ^a^	6.98 ± 0.53 ^b^	6.06 ± 0.33 ^ab^

Values are expressed as means ± S.E. (n = 8). ^abc^ Different letters indicate significant differences among experimental groups (*p* < 0.05). HbA_1c_, glycosylated hemoglobin; Ca, calcium; IP, inorganic phosphorus; NC, normal control group; DM, diabetic control group; DM+BP, diabetic-bergapten group; DM+MTS, diabetic-methoxsalen group.

**Table 2:** The effects of bergapten or methoxsalen supplementation on bone morphometry and microstructure parameters in diabetic mice.

	NC	DM	DM+BP	DM + MTS
**Femoral morphometry**				
Length (mm)	15.63 ± 0.11	15.37 ± 0.16	15.58 ± 0.35	15.17 ± 0.21
Weight (mg)	83.89 ± 1.57 ^b^	73.11 ± 3.48 ^a^	81.57 ± 3.07 ^b^	80.35 ± 1.93 ^ab^
**Femoral trabecular bone microstructures**				
BV/TV (%)	32.00 ± 2.59 ^b^	19.63 ± 2.34 ^a^	30.14 ± 1.93 ^b^	26.90 ± 1.70 ^b^
Tb.N (mm^−1^)	2.55 ± 0.13 ^b^	1.81 ± 0.17 ^a^	2.51 ± 0.16 ^b^	2.44 ± 0.09 ^b^
Tb.Th (μm)	124.17 ± 3.75 ^c^	107.10 ± 2.73 ^a^	120.64 ± 4.40 ^bc^	109.79 ± 4.50 ^ab^
Tb.Sp (μm)	223.76 ± 9.69	262.87 ± 15.97	237.70 ± 20.27	254.00 ± 14.10
SMI	2.02 ± 0.10 ^a^	2.39 ± 0.08 ^b^	1.98 ± 0.09 ^a^	1.99 ± 0.06 ^a^
**Tibial morphometry**				
Length (mm)	18.16 ± 0.13	18.07 ± 0.10	18.21 ± 0.08	18.28 ± 0.34
Weight (mg)	60.04 ± 1.61	55.50 ± 1.34	60.36 ± 2.82	59.47 ± 1.85
**Tibial trabecular bone microstructures**				
BV/TV (%)	31.26 ± 2.52 ^bc^	20.82 ± 2.88 ^a^	34.57 ± 3.17 ^c^	24.66 ± 3.10 ^ab^
Tb.N (mm^−1^)	2.67 ± 0.13	2.12 ± 0.22	2.77 ± 0.22	2.15 ± 0.17
Tb.Th (μm)	116.12 ± 4.44 ^b^	96.43 ± 3.90 ^a^	123.77 ± 3.30 ^b^	112.52 ± 8.36 ^b^
Tb.Sp (μm)	222.28 ± 7.68	234.95 ± 13.08	218.12 ± 19.93	246.71 ± 10.74
SMI	2.19 ± 0.11 ^ab^	2.41 ± 0.10 ^b^	1.96 ± 0.14 ^a^	2.37 ± 0.05 ^b^

Values are expressed as means ± S.E. (n = 8). ^abc^ Different letters indicate significant differences among experimental groups (*p* < 0.05). BV/TV, bone volume density; Tb.N, trabecular number; Tb.Th, trabecular thickness; Tb.Sp, trabecular separation; SMI, structure model index; NC, normal control group; DM, diabetic control group; DM+BP, diabetic-bergapten group; DM+MTS, diabetic-methoxsalen group.
